# Living beyond placenta accreta spectrum: parent’s experience of the postnatal journey and recommendations for an integrated care pathway

**DOI:** 10.1186/s12884-022-04726-8

**Published:** 2022-05-10

**Authors:** Helena C. Bartels, Antje Horsch, Naomi Cooney, Donal J. Brennan, Joan G. Lalor

**Affiliations:** 1 Dept of UCD Obstetrics and Gynaecology, School of Medicine, University College Dublin, National Maternity Hospital, Holles Street, Dublin 2, Ireland; 2 Institute of Higher Education and Research in Healthcare, University of Lausanne, Lausanne, Switzerland; 3 Woman-Mother-Child Department, Lausanne University Hospital, Lausanne, Switzerland; 4 Placenta Accreta Ireland, Patient Support and Advocacy Group, Dublin, Ireland; 5 School of Nursing and Midwifery, Trinity College Dublin, Dublin 2, Ireland

**Keywords:** Placenta accreta spectrum, Qualitative, Interview, Experience, Trauma, Multi-disciplinary team care, Postnatal care

## Abstract

**Background:**

Placenta Accreta Spectrum is associated with significant clinical maternal morbidity and mortality, which has been extensively described in the literature. However, there is a dearth of research on the lived experiences of pregnant people and their support partners. The aim of this study is to describe living beyond a pregnancy and birth complicated by PAS for up to four years postpartum. Participants experiences inform the development of an integrated care pathway of family centered support interventions.

**Methods:**

An Interpretative Phenomenological Analysis approach was applied to collect data through virtual interviews over a 3-month period from February to April 2021. Twenty-nine participants shared their stories; six people with a history of PAS and their support partners were interviewed together (*n* = 12 participants), six were interviewed separately (*n* = 12 participants), and five were interviewed without their partner. Pregnant people were eligible for inclusion if they had a diagnosis of PAS within the previous 5 years. This paper focuses on the postnatal period, with data from the antenatal and intrapartum periods described separately.

**Results:**

One superordinate theme “Living beyond PAS” emerged from interviews, with 6 subordinate themes as follows; “Living with a different body”, “The impact on relationships”, “Coping strategies”, “Post-traumatic growth”, “Challenges with normal care” and recommendations for “What needs to change”. These themes informed the development of an integrated care pathway for pregnant people and their support partners to support them from diagnosis up to one year following the birth.

**Conclusion:**

Parents described the challenges of the postnatal period in terms of the physical and emotional impact, and how some were able to make positive life changes in the aftermath of a traumatic event. An integrated care pathway of simple supportive interventions, based on participant recommendations, delivered as part of specialist multidisciplinary team care may assist pregnant people and their support partners in alleviating some of these challenges.

## Introduction

Placenta Accreta Spectrum (PAS) is a condition where a defect of the decidua basalis, the base of the placental bed, results in varying degrees of placental adherence and invasion [1]. As a result, the placenta fails to separate after the birth. Understandably, much of the literature to date has focused on the associated maternal morbidity and mortality, which is largely related to massive obstetric hemorrhage [2–4]. The psychological sequalae of PAS have received relatively less attention and only a small number of publications have begun to explore this [5–7].

Two qualitative interview studies have begun to uncover the maternal lived experience of PAS, with one study (*N* = 7) describing pregnant peoples’ feelings of guilt, fear and worry which remained long after the birth [6], with the second study (*N* = 17) revealing the significant emotional burden and uncertainty a diagnosis of PAS may bring [7].

Furthermore, pregnant people with PAS are more likely to report anxiety/worry, grief and depression compared to those after a caesarean birth complicated by other factors [8]. A survey study exploring general quality of life in the postnatal period found the mental health impacts of PAS were long lasting, with mental health score domains persistently low in the months after the birth, even as physical health improved [9].

While these studies have started to describe the pregnant persons’ experience of a pregnancy complicated by PAS, the support partner’s perspective is currently absent from the literature.

The purpose of this study is to present the lived experience of recovering from a pregnancy complicated by PAS and experiences of care as told by pregnant people with PAS and their support partners. Their recommendations inform a suggested integrated care pathway to support pregnant people and their support partners from the time of receiving the diagnosis to long term postnatal care.

## Methods

This was a qualitative study using an Interpretative Phenomenological Analysis (IPA) approach. This paper presents data relating to the postnatal journey, with the antenatal journey and the birth described separately and still under study.

IPA was chosen as the most appropriate methodology because the aim of the study was to explore a specific phenomenon, in this case to explore mothers’ and fathers’ lived experience of PAS [10, 11]. Interviews allowed mothers and fathers to describe their personal experiences of what it is like to live life beyond a diagnosis of PAS, inviting them to describe their experiences of specialist team care and to consider ways in which care could be improved.

Participants were recruited through a patient advocacy group Placenta Accreta Ireland. The study design and confirmation of themes was conducted in collaboration with this group, with feedback sought throughout the research process. An initial meeting was held between advocacy group Placenta Accreta Ireland and the researchers to introduce the project and to seek input from key stakeholders. People were eligible for inclusion when they had experienced a pregnancy complicated by PAS within the past five years, had sufficient English language skills, and were able to consent and aged over 18 years. Support partners were eligible when their partners had met the above inclusion criteria, had sufficient English language skills and were able to consent. Where eligible, previously pregnant people and/or their partners expressed an interest to participate in the study, a patient information leaflet outlining the study was sent to them, with time given to consider their participation. Study participation was confirmed by written consent. They were given the choice to either be interviewed together or separately.

Medical records of participants were reviewed to obtain selected clinical outcome data such as antenatal diagnosis of PAS, delivery outcomes and histological confirmation of PAS.

Interviews were conducted virtually over a three-month period between February and April 2021 by three researchers (HB (MD), JL (PhD), AH (PhD)) and were recorded using the audio function in Zoom (Zoom Video Communications Inc. 2016) with participant consent and were transcribed using Sonix (Sonix, Inc. San Francisco, 2021,). To ensure patient confidentiality, all audio files and transcripts were pseudonymized and allocated a unique study identifier (M = Mother, F = Father). Consent forms were stored securely on a separate secure server and cannot be linked to the transcripts or audio files.

Themes were developed using an IPA approach [12, 13], with procedures described by IPA [14, 15] followed throughout the analysis to ensure methodological rigour and credibility. Rigour throughout the analytic process was ensured in the following ways; an initial analysis of participant transcripts was performed by the researcher who conducted the interview followed by confirmation and validation of themes performed at regular meetings within the research team. Data saturation was defined as an absence of new themes arising from subsequent interviews. This was reached by the conclusion of the study. Peer validation was performed with two additional research team members (NC, DB) who had not been involved in data collection to ensure credibility of the emerging themes.

Furthermore, the themes developed from this study were shared with non-participant members of Placenta Accreta Ireland, who confirmed that they resonated with their own experiences. The development of the integrated care pathway of family centered support interventions was co-created based on the data with members of Placenta Accreta Ireland to ensure that the pregnant person and their support partner remained central to the care given. Co-creation of an integrated care pathway allowed us to involve key stakeholders in the recommendations made, where both those who provide, and avail of the service are actively involved to ensure the pathway keeps the pregnant person and their support partner at the center of care [16, 17].

The data presented here relates to the postnatal journey of PAS, although participants also described their experience of the antenatal journey and the birth using the same methodology as described here. The data relating to the antenatal journey and the birth are described and published separately. The integrated care pathway presented here incorporates recommendations made by participants on the postnatal journey as described in this study, and recommendations relating to the antenatal journey and the birth.

All participants identified themselves as mothers or fathers, hence when reporting on the experiences of the participants they were referred to as mothers and fathers. However, we recognize that there are many ways in which parents identify and that heteronormative stereotypes are not always applicable or relevant.

Ethical approval was granted by the hospital ethics committee and written informed consent was obtained from participants (ref EC25.2020).

## Results

This study included 29 participants, consisting of six mothers with a history of PAS and their support partner who were interviewed together (*n* = 12), six couples where the mother and their support partner were interviewed separately (*n* = 12), and five mothers who were interviewed without their partner. Table 1 provides a summary of participants demographics. Participants were between 3 months and 4 years after the birth at the time of the interviews and all had at least one prior caesarean birth. All mothers except one were antenatally diagnosed with PAS, 16 underwent total caesarean hysterectomy and the median (IQR) length of stay in hospital postnatally was 6 days (4–9). One participant underwent uterine conservation surgery with myometrial resection.

**Table 1:** Participant demographics and selected clinical outcomes

Characteristics of pregnant people	Participants (*n* = 17)
Age	37 (34–43)
Gestation at diagnosis (weeks)	23 (20–28)^a^
Gestation at time of birth (weeks)	33 + 6 (29 + 5—35)
Emergency delivery n (%)	7 (42)
Mode of anaesthesia	
Primary neuroaxial	4 (23)
General anaesthesia	7 (42)
Neuroaxial with conversion to general anaesthesia	6 (35)
Hysterectomy n (%)	16 (94)
Length of NICU admission (days)	25 (15–40)
Time since PAS pregnancy (months)	18 (12.5–21.5)

^a^One participant undiagnosed during pregnancy

Data presented as median (IQR) unless otherwise stated

There was one superordinate theme of the postnatal period “Living beyond PAS”, with six subordinate themes emerging – “Living with a different body”, “The impact on relationships”, “Coping strategies”, the challenges of normal care “Lack of specialist review”, “Post-traumatic growth” and “What needs to change”.

### Superordinate theme: Living beyond PAS

Participants described the challenges they faced in endeavoring to live beyond PAS. Many related to the physical recovery associated with major surgery; however, the emotional impact of surviving a birth with a significant risk of maternal mortality took its toll. A perception of uncontrolled pain, a changing identity and irrevocable infertility were but some of the challenges faced. Additional supporting quotes for the postnatal journey are provided in Table 2.

**Table 2:** The postnatal journey

Superordinate theme: “Living beyond PAS”	
**Subordinate themes**	**1. Living with a different body**
	*Mother: “When they tell you all this stuff at the start, you're like, I'll do anything. You know, I just want my baby and whatever to go to the plan and I'll do whatever they say. At the time it's all about this baby, this pregnancy. But then it's not as easy as you think after the hysterectomy, so that's that part of my life over.” (PAS01M)*
	*Mother: “I think I'm on the right path, but it's still hard and I still kind of find there’s not much you can do to help your tummy, I feel like a hard lump right at my belly. I don't like the feel of it. Like I know I won’t don't go around in shorts or anything like that!” (PAS03M)*
	*Mother: “It’s upsetting because we’ll never be able to do this again, I got rid of my maternity clothes rather than keep them just in case. I have zero libido, I look disfigured. I just try to not be conscious because, you know, again, I'm lucky to have my two babies and everything else, but definitely affects me in terms of how I feel about myself and moving forward with everything” (PAS05M)*
	*Mother:* “*I would never have had sex again if it was left behind I’d be so worried, it took about six months to get back to normal” (PAS16M)*
	*Mother: “It took me a long time to come to terms with having a hysterectomy…. I haven’t admitted to friends I had a hysterectomy. I don't know why, but I'm not ashamed of it. It's just, you know, because I kind of find when you tell people you get I think it is just because you just get a real pity party when you do tell people and that doesn't do anything.” (PAS06M)*
	*Mother: “I can talk about the accreta but I stop at the hysterectomy part…they happen to older women…it’s the stigma. I didn’t feel like a woman anymore.. I knew nothing about this- one day you’re fine and the next day it’s all taken from you…He didn’t know this was happening…. So does that mean I can’t have kids anymore? I’m still in denial” (PAS09M)*
	*Mother: “I was so young to have a hysterectomy. I actually was a bit more upset about that. And I was embarrassed. I was really embarrassed saying that to people” (PAS21M)*
	**2. Impact on relationships**
	*Mother: “People expect you to just forget everything, seeing your baby and saying “it’s all worth it”, once you’re home people thing you’re grand…I’m still going through this now. I don't think we're at the point that we would even survive a discussion about it. I think we just need to kind of get better. He's moved on because he had to move on.” (PAS03M)*
	*Mother: “And I just needed that space and to heal and even just be away from people” (PAS21M)*
	*Father: “It's over. It's done. I just don't like to dwell too much on this because I get stuck kind of unhappy. I think you're trying to deal with the difficult issue which brought us closer. I'm not very good at talking in general about anything that's serious.” (PAS01F)*
	*Father: “We don’t talk about it now…it all just comes back” (PAS04F)*
	*Father: “Its tough road (she) has been down she has had flashbacks…I didn’t have time to think about it, I just had to be strong… the good days are few and far between” (PAS10F)*
	*Father: “… it was traumatic. It was awful what we went through. But you just got to get on with it and make the best of a bad situation. She was carrying the trauma around, what she had gone through…like an elephant she keeps coming back to things… I think you have to put it away…I think it threatened us as she wasn’t coming to terms with it”. (PAS15F)*
	**3. Coping strategies (postnatal)**
	*Mother: “I'm just like using whatever coping mechanisms I can, like, you know, I used to listen to music, I used to sing in a lot of choirs when I was younger and I used to listen to, like, this particular choral work. And it just kept me calm. And I would just like focus on that and just listen to that and just keep calm” (PAS06M)* *Mother: “I would never have done it without my mom. I have to say, my mom was amazing. She came every day and helped me and, you know, cooked me dinner. So I don't know how anyone can do it without having that extra support” (PAS21M)*
	*Mother: “So just even to talk to people on the phone or to see them when I was feeling up to it, that helped. And also the kids, you know, the smiles on their faces, that will help” (PAS20M)*
	**4. Posttraumatic growth**
	*Mother: “I was very lucky because that bond has been amazing through all of this. And I think it's made me just very, very grateful for being well now. And I feel very lucky now, I swim. I don't think I'd ever thought I'd be able to do what I can now back then.” (PAS21M)*
	*Mother:* “*Yeah, and I'm stronger than I thought I was. So I'm just so grateful every day to have my baby and to have my own life and for health” (PAS20M)*
	*Mother: “It definitely humbled me. So many people get caught up in this rat race of trying to get bigger and better all the time. I do not take anything for granted now, I have more faith now. So it really humbled me in many ways.” (PAS19M)*
	*Father: “Like I would say, we're both huge changed, you know? Changed much for the better” (PAS02F)*
	*Father: “*… *I want to be able to look back at baby pictures and say how far we have come not to look and be thrown back into that fear… I didn’t want her to worry about me or for her to see me break”. (PAS23F)*
	**5. Challenges with normal care “lack of specialist review”**
	*Mother: “I got a letter reminding me to get a smear, but I have no cervix….” (PAS06M)*
	*Mother: “But nobody tells you how to cope with that afterwards. It's not like not only on the cosmetic end of it, but like the practical side of it, like, you know, the jeans will be uncomfortable…” (PAS19M)*
	*Mother: “I find it hard to believe you are never looked at again” (PAS22M)*
	*Father: “We needed more medical back up in the early weeks.. we didn’t know where to get help. … it was like having a sick person back in the house, not like someone who has just given birth. You think she’ll bounce back, but this is different.” (PAS17F)*
	**6. What needs to change**
	*Mother: “I think that would be helpful, that kind of general information, you know, that everyone could read or that you could just hand somebody that that would be really good” (PAS 21 M)*
	*Mother: “There was no debriefing…no specialist review. There needs to be more follow up, recommend 6 week, 6 month and 12 month follow up … it’s so rare that it’s challenging to find information” (PAS09M)*
	*Mother: “And women who have a complicated delivery subjected to the standardized normal delivery, I think there should be more and more of training in how they care for women who've gone through hysterectomy. The standard care does not apply to us!” (PAS18M)*
	*Father: “Follow up 6 weeks, months one year and build in counselling … should be a standard level of aftercare… shouldn’t be cut off…. Specialist care (level of knowledge)… lack of sources of information. It was difficult,I probably wasn’t going to go for counselling. Men need to be encouraged to get support” (PAS08F)*
	*Father: “*… *she had to have some counselling I think it helped a lot… there could have been a lot more information. Every man is so different… some may open up? Men struggle to open up…give them more information its quite a traumatic experience.” (PAS15F)*
	*Father: “I was doing some research trying to find out about placenta accrete and there didn't seem to be like a lot of information readily available that was quite clear and concise, so it was a learning curve.” (PAS17F)*

### Subordinate theme 1: Living with a different body

Undergoing caesarean hysterectomy had a profound, long-lasting impact on mothers’ physical and emotional health. The physical toll of their experience of PAS was never far from their minds, with a large abdominal scar acting as a constant visual reminder. Some mothers described their bodies post-surgery as disfigured. This was amplified by perceived long lasting physical pain both in hospital and following discharge home. The impact of this prolonged perceived pain negatively impacted on their quality of life and on activities of daily living, as simple tasks such as wheeling their baby in the pram, became unmanageable. This loss of independence left many feeling as though they were a burden on others. For fathers, witnessing their partner in pain was difficult, with some describing feelings of helplessness and unsure how best to support or advocate for their partner. One father said “*She was in terrible pain…, if I had been told what to look out for I could have been more vocal..” (PAS08F),* highlighting the need for clear pre-operative counseling and communication regarding post-operative pain in these cases.

All but one of the participants had a caesarean hysterectomy, and the loss of fertility affected participants differently, depending on whether they had living children and if they felt their family was complete. Some described the loss of fertility as a loss of womanhood as they could no longer bear children*. “I don’t need my uterus anymore, it shouldn’t bother me but it does…I struggle with it….I am still breastfeeding and I’m reluctant to give it up as that’s my only link to motherhood now (without my womb)..I no longer have a connection to being able to have babies…it affected how I feel about myself as a woman….” (PAS05M).* For two participants in particular, the loss of fertility was devastating; one pregnant person and their support partner who had no living children, while another had one child together and had intended to have more. In contrast, for other participants, the inability to have further children came as a relief and closed the door on future pregnancies that could also be complicated by PAS, an event they would feel unable to go through again, one mother said*: “I definitely have no desire to have another baby… your body’s not able to do it…I couldn’t have gone through that again” (PAS11M)”.* Similarly, fathers placed less importance on the loss of fertility than their partners, even when the family was incomplete, as a further pregnancy was seen to be too much to endure*; “I didn’t want a football team, I don’t want any more kids.. I didn’t want more after number two- why push our luck? I didn’t want to go through all that again” (PAS15F).*

Some mothers described feelings of shame and stigma associated with having a hysterectomy. For many, they were embarrassed as young people to have undergone this procedure, as they associated a hysterectomy with older people. *“I was really embarrassed saying that to people from the minute I heard it and then having to tell anybody” (PAS21M).* Many participants were immensely frustrated at the lack of evidence-based information relevant to young people having a hysterectomy, as much of the information available is targeted at older people undergoing hysterectomy for uterine prolapse. “*There’s very little information about younger women having a hysterectomy….it was a constant frustration….it’s hard to get supports because it’s such a rare thing….” (PAS06M).*

### Subordinate theme 2: Impact on relationships

As mothers adapted to life after PAS, the impact on the relationships in their life was evident. Some participants felt a stronger bond having survived a shared traumatic experience together: *“dealing with a difficult pregnancy has brought us closer” (PAS01F)*. For others, the relationship between partners deteriorated, the root cause of which varied for mothers and fathers. For mothers, they felt unable to discuss the pregnancy with their partner for fear it might evoke a response such as *“for the survival of our relationship we just need to get on with things, I don’t think we would survive a discussion about it” (PAS05M).* On the other hand, fathers felt their partner needed to move on from the pregnancy and discussion around the trauma they experienced would not be helpful: *“… I had to provide for the family… It was awful what we went through…but you just got to get on with it and make the best of a bad situation.” (PAS15F).* This left a gap between what each member of the couple needed and for some became “a taboo topic”. Furthermore, the impact on participants’ physical relationship was significant. Mothers reported a loss of libido, vaginal dryness and feeling unattractive in a disfigured body as barriers to the return of sexual intimacy. One woman said “*it took about six months to get back to normal* (vaginal dryness)*” (PAS16M)*. Fathers often felt their partner had no interest in sex due to perceived physical pain “*(she) was very tender for a long time, she could just about manage steps.. even her clothes hurt her” (PAS08F).*

### Subordinate theme 3: Coping strategies

Parents reflected on the coping strategies they relied on in the postnatal period. Support from friends and family was central; however, it was not available to everyone. Mothers reflected on the importance of keeping in touch with friends and enjoying time with their children. This was shared by fathers, who were grateful to have a healthy partner and infant after the threat PAS had posed to their survival. Some mothers used meditation to calm themselves when they felt themselves becoming anxious: *“I step back and I breathe and I try and engage my brain to think rationally, like, I mean, and. I just try and just calm myself a bit” (PAS20M).* Other mothers reflected on how helpful being part of Placenta Accreta Ireland advocacy group had been, emphasizing how the group provided them with people to talk to who understood the journey they had been on and the recovery they were still going through. This was shared by fathers, who reported seeing the benefit for their partner of being part of the group *“…..the sessions with the support group were really good…..really helped her to talk about it” (PAS04F).* Both mothers and fathers emphasized the importance of mental health support and counselling to navigate the emotional challenges of recovering from PAS. Fathers in particular felt they were not offered any counselling or mental health support, and didn’t feel empowered to ask for help; however, if they had been, some indicated they would have needed encouragement to attend: “*…I probably wasn’t going to go for counselling (she did attend)… men need to be encouraged to get support” (PAS08F).*

### Subordinate theme 4: Post-traumatic growth

Participants shared various ways in which they experienced post-traumatic growth, with a strengthening of the relationship with their partner, a feeling of extreme gratitude, living in the moment and wanting to help others as central factors. Fathers reflected on the bond with their partner and being in awe of their resilience in bearing all they had to endure for their family “*I suppose we have got closer, we have appreciated more for what each of us have done, especially for what she has gone through, you know, takes a lot of courage” (PAS19F).* Those who experienced post-traumatic growth reflected on how they didn’t know their own strength, and how they had grown as people, describing themselves as changed after what they had experienced*.* Gratitude at having survived was evident, even though participants were facing ongoing challenges in their physical and emotional recovery. Mothers felt very grateful to have survived *“And I think it's made me just very, very grateful for being well now” (PAS21M)* with fathers being grateful for the survival of both their partner and baby *“…Felt I was even though I'd been through a lot of trauma, I was so grateful as well that they survived and that she survived it and that they were coming home….so I'm just so, so grateful to this day” (PAS20F).*

This new-found sense of gratitude made participants want to make the most of everyday and they expressed a sense of ‘living in the moment’, something they had failed to appreciate before PAS. Furthermore, they wanted to give back and make things better for others going forward *“and we have more empathy, I think, and we both are passionate about how do we give back and how we help others.” (PAS02F).*

### Subordinate theme 5: Challenges with normal care “lack of specialist review”

Participants shared the frustration and difficulty of being cared for as if they had had a routine caesarean birth in the postnatal period and how this was unsatisfactory in meeting their needs, both immediately after birth and in the long term. This was in stark contrast to the specialist care received in the antenatal period, which one mother described as *“I couldn’t believe how they looked after me…I felt like their family…it was incredible how we were treated (PAS01M)”.* Mothers described being reviewed by healthcare staff who expected them to be recovering at a pace consistent with a straightforward caesarean birth, and not always being met with understanding as to why it would be difficult to hold their baby, why they would need additional pain relief or might be struggling emotionally after a traumatic experience: *“There's almost a disconnect, though, between like the physio who deals with all the pregnant women and then tells them all the same thing…things they recommend are not really feasible….(PAS01M)”* and the doctors caring for them *“I definitely think the care fell apart* [after the birth] *….before* [the birth] *the care was great, afterwards it fell apart” (PAS04M).* Some participants reported receiving reminders for cervical screening and being asked about contraception although they had had a caesarean hysterectomy, one mother said “*The normal pack doesn’t apply to us… they were going to give me information on contraception and said …sure you won’t need that…they mentioned cervical check and said I don’t know if you’ll need that …” (PAS10M).* Participants were unanimous in their call for additional follow-up and the difficulty they faced by having no opportunity to meet with their specialist team. Fathers described attending for follow-up with their partner and being seen in a standard outpatient clinic where no member of the specialist team was present who knew them or could appropriately debrief them. There was a sense of helplessness, as mothers and fathers wanted to engage with the hospital but did not have a point of contact or know how to get the follow-up they were seeking. Where mothers were reviewed, this was often limited to a once off 6-week postnatal check where the focus was very much on physical recovery, rather than how they may be coping emotionally. Furthermore, mothers commented that often at 6 weeks they had not even themselves had the opportunity to process the experience, as for many, pain was still an issue, and for some their baby was still in NICU or only recently discharged home. Consequently, mothers expressed a need for a further follow-up appointment to review their physical and emotional recovery after they had time to process how they feel about this traumatic event.

### Subordinate theme 6: What needs to change

Throughout participant interviews, it became evident that mothers and fathers had many suggestions and recommendations on how care could be improved for others going forward. Recommendations were simple and were focused on three distinct episodes of the PAS experience, the antenatal journey, the birth, and the postnatal journey.

Antenatally, parents voiced concerns as to the lack of reliable, high-quality information available to those with PAS. Some struggled to find evidence-based resources to inform themselves about the condition: “*I was doing some research trying to find out about accreta … there didn't seem to be like a lot of information readily available that was quite clear and concise” (PAS17F).* Participants called for the provision of information leaflets, something tangible they could take home with them, which had been reviewed by their interdisciplinary team and was therefore reliable.

In their recovery after the birth, mothers struggled with deconditioning of their physical health and called for input from physiotherapy during pregnancy. Mothers who had an extended hospital stay during pregnancy advised they would have benefited from physiotherapy review while they awaited the birth to prepare, not just for birth, but how to move and navigate their body after the surgery. Mothers suggested an antenatal consultation with a physiotherapist was important so they could be introduced to the exercises, what to expect from their physical body after birth and have the tools to help themselves during recovery.

On the day of the birth, fathers advocated for dedicated time to spend with their partner before going to theatre *“I never got to say goodbye or tell her I love her….she was just gone…I was just left sitting there” (PAS8F)*. Some fathers felt like bystanders during the pregnancy and particularly on the day of the birth and called for a contact person that could keep them updated while their partner was in theatre.

Around the birth and postnatally, pain management was a challenge for some mothers. Participants called for standardized pain relief to be available that met the needs of the extensive surgery they had been through, staff to be receive education and training on PAS aftercare and when to administer pain relief, and that the provision of pain relief should be provided at regular intervals. Furthermore, many mothers had to be admitted to hospital for many weeks and called for simple infrastructural changes to improve the stay for long-stay mothers, such as new mattresses and heating.

**Fig. 1:**
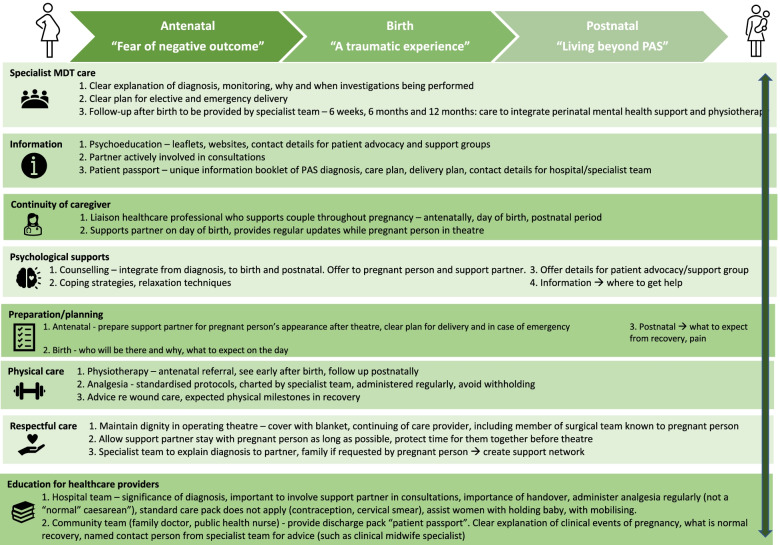
Family Centered support interventions

These recommendations informed the development of a suggested integrated care pathway of family centered support interventions, shown in Fig. 1. The pathway describes a proposed, integrated care pathway which healthcare providers can refer to when caring for mothers with PAS and their families. The pathway encompasses the recommendations of participants and supports at each step of care they hope future families could avail of, from diagnosis to the postnatal period.

## Discussion

This study presents the lived experience of PAS as told by mothers and fathers. Interviews revealed the lasting impact of a traumatic pregnancy, the coping strategies they implemented, and, for some, the personal growth gained in the aftermath of this shared traumatic experience. Recommendations made by participants highlight the need to provide family-centered care, to include pre-operative preparation, access to reliable information, continuity of care from a specialist team, involving partners in all stages of care and ensuring regular follow-up is in place after the pregnancy. It appears simple interventions such as those recommended in the care pathway may improve the pregnancy experience for pregnant people with PAS and their support partners and support them as they try to cope with the fear and uncertainties described.

This study supports the findings of previous research in this area and explores how healthcare providers can improve the experience for pregnant people with PAS. The recommendations are modest and can realistically be introduced along with specialist MDT care by healthcare providers.

An interesting finding from this study were the multiple ways in which pregnant people with PAS and their support partners endeavored to make positive life changes in the aftermath of a perceived traumatic event. As participants perceived PAS as a life-threatening event, it undoubtedly can be described as a traumatic event [18]. It is well recognised that traumatic events can lead to strengthening and growth, a phenomenon described as post-traumatic growth [19, 20]. Suffering a life crisis, such as a highly complex pregnancy, can lead to constructive changes in individuals, as they attempt to cope in the aftermath of such an event [21]. This was evident from some of the participant interviews, as the trauma of the pregnancy allowed some to set new life priorities, have an increased appreciation for life, and a recognition of their own strength and resilience. Research has demonstrated that both good coping strategies and strong social support networks are important factors, which increase the likelihood that individuals will experience post-traumatic growth after life changing events [14, 15, 22].

Mothers and fathers may therefore benefit from strategies implemented during pregnancy to support them to develop mature coping strategies. Coping strategies aimed at managing fear and uncertainty during pregnancy and preparing for what to expect during recovery could provide them with the tools to allow for post-traumatic growth in the aftermath of a pregnancy complicated by PAS [23]. Furthermore, the fact that those with a strong social support network are more likely to find strength in the aftermath of trauma demonstrates the importance of this social support. Many participants described the reliance on friends and family as essential to cope. However, for some participants this social network was not available, with participants citing a lack of understanding of what they had experienced and therefore not recognising their need for additional support as being major factors. Hence, increased education and awareness around PAS and its physical and emotional sequalae would likely be of benefit in providing this missing knowledge to family members and friends of those impacted by PAS.

Following on from this, it was clear from interviews that counselling and preparation during pregnancy of what to expect from the birth and recovery is essential. This counselling should be provided to pregnant people and their support partners together, as it was evident from interviews that fathers felt ill prepared to support their partner in the postnatal journey, in particular relating to issues such as post-operative pain, expected pace of recovery and loss of fertility. Numerous studies have described the importance of pre-operative counselling and education prior to surgery for other indications, which has been shown to reduce anxiety, pain and fatigue post-operatively, and forms an essential part of ERAS protocols [24–27]. Hence the benefits of pre-operative counselling are well described and would likely assist pregnant people and their support partners to manage their expectations in recovery.

Finally, a strong theme was the lack of specialist care in the postnatal period. Participants were vocal in their praise of the specialist care they received during pregnancy but felt this “fell apart” after birth. It is clear this needs to change and a pathway for follow-up implemented. Integrated within the care pathway should be education of healthcare providers to highlight the importance of follow up care. Participants struggled to find information on recovery and knowing what is normal, and it seems apparent that the offer of regular follow-up, perhaps up to 12 months postnatally, would assist in alleviating some of these uncertainties. Certainly, this is strongly recommended by all in this study.

This study has several strengths and limitations. We present for the first time the voice of the father and their unique perspective on what it is like to be a partner of someone with PAS, which allowed researchers to gain a very in-depth view of how pregnant people with PAS and their support partners navigate their pregnancy and recovery. Each step – from study design, recruitment, data collection, and analysis of themes—was conducted with public and patient involvement (PPI) in the research process through liaison with a patient advocacy and support group.

The most significant limitation to the study is that all participants were recruited through their membership of Placenta Accreta Ireland, and so there are no data from pregnant people and their support partners who do not avail support in this way. However, the findings presented here are similar to previous studies undertaken with mothers with PAS [6, 7]. Furthermore, the participants in this study had an earlier median gestational age at delivery, likely as a result of the rate of emergency deliveries in the cohort prior to 34 weeks, as well as a high rate of caesarean hysterectomy, which may influence the experience of participants. However, previous work has not shown that pregnancy outcomes such as emergency delivery or hysterectomy are associated with decreased quality of life in PAS [9].

In conclusion, pregnant people and their support partners who are living beyond a pregnancy complicated by PAS may face a long recovery, both physical and emotional. Some are able to use this traumatic event for positive change in their lives. Recommendations made highlight the need to provide family-centred care, with a particular focus on continuity of care throughout pregnancy and the postnatal period, involving support partners in discussions and ensuring regular follow-up is in place after the birth. Future research should focus on the factors which improve the pregnancy experience for those with PAS and their support partners and evaluate the implementation of the recommendations included here. Some of these key recommendations are described in Fig. 1, a family-centered care pathway of supportive interventions to assist those affected as they navigate a pregnancy and recovery complicated by PAS.

## Data Availability

Data obtained for this study was in the form of interview transcripts from participants. These are not publicly available or upon request from the authors to protect patient confidentiality. Sample quotes which support the themes presented within the manuscript are shared within the text and supporting tables.

## Abbreviations

PAS: Placenta accreta spectrum
MDT: Multi-disciplinary team
